# Outcomes of primary versus conversional Roux-En-Y gastric bypass after laparoscopic sleeve gastrectomy: a retrospective propensity score–matched cohort study

**DOI:** 10.1186/s12893-024-02374-7

**Published:** 2024-03-06

**Authors:** Mohamed Hany, Bart Torensma, Ahmed Zidan, Mohamed Ibrahim, Anwar Ashraf Abouelnasr, Ann Samy Shafiq Agayby, Iman El Sayed

**Affiliations:** 1 Department of Surgery, Medical Research Institute, Alexandria University, 165 Horreya Avenue, Alexandria, 21561 Egypt; 2 Consultant of bariatric surgery at Madina Women’s hospital (IFSO-certified bariatric center), Alexandria, Egypt; 3 Clinical Epidemiologist, Leiden University Medical Center (LUMC), Leiden, The Netherlands; 4 Biomedical Informatics and Medical Statistics Department, Medical Research Institute, Alexandria University, Alexandria, Egypt

**Keywords:** Primary roux-en-Y gastric bypass, Conversional Roux-en-Y gastric bypass, Laparoscopic sleeve gastrectomy, Bariatric surgery, Food tolerance, Weight loss failure, Gastroesophageal reflux disease

## Abstract

**Background:**

Conversional surgery is common after laparoscopic sleeve gastrectomy (LSG) because of suboptimal weight loss (SWL) or poor responders and gastroesophageal reflux disease (GERD). Roux-en-Y gastric bypass (RYGB) is the most common conversional procedure after LSG.

**Methods:**

A retrospective cohort study analyzed patients who underwent primary RYGB (PRYGB) or conversional RYGB (CRYGB) at three specialized bariatric centers between 2008 and 2019 and tested for weight loss, resolution of GERD, food tolerance (FT), early and late complications, and the resolution of associated medical problems. This was analyzed by propensity score matching (PSM).

**Results:**

In total, 558 (PRYGB) and 155 (CRYGB) completed at least 2 years of follow-up. After PSM, both cohorts significantly decreased BMI from baseline (*p* < 0.001). The CRYGB group had an initially more significant mean BMI decrease of 6.095 kg/m^2^ at 6 months of follow-up (*p* < 0.001), while the PRYGB group had a more significant mean BMI decrease of 5.890 kg/m^2^ and 8.626 kg/m^2^ at 1 and 2 years, respectively (*p* < 0.001). Food tolerance (FT) improved significantly in the CRYGB group (*p* < 0.001), while CRYGB had better FT than PRYGB at 2 years (*p* < 0.001). A GERD resolution rate of 92.6% was recorded in the CRYGB (*p* < 0.001). Both cohorts had comparable rates of early complications (*p* = 0.584), late complications (*p* = 0.495), and reoperations (*p* = 0.398). Associated medical problems at 2 years significantly improved in both cohorts (*p* < 0.001).

**Conclusions:**

CRYGB is a safe and efficient option in non- or poor responders after LSG, with significant weight loss and improvement in GERD. Moreover, PRYGB and CRYGB had comparable complications, reoperations, and associated medical problem resolution rates.

**Supplementary Information:**

The online version contains supplementary material available at 10.1186/s12893-024-02374-7.

## Background

Laparoscopic sleeve gastrectomy (LSG) as a stand-alone weight loss procedure has shown increased popularity over the last decade, becoming the most commonly performed bariatric metabolic surgery (BMS) worldwide in 2014 and constituting more than half of all BMS worldwide in 2018 because of its high safety profile and satisfactory short-term outcomes [1, 2]. On the other hand, conversional surgery for the suboptimal effect of the LSG has become a well-known issue that seems inevitable in some patients after LSG, with a prevalence of 2.5–30% post-LSG in the literature [2]. Poor responders with suboptimal weight loss (SWL) are the leading reported causes of conversional after LSG, constituting nearly 70% of conversional after LSG, followed by gastroesophageal reflux disease (GERD), and dysphagia [2–11]. Moreover, some authors reported increasing conversional rates with time, ranging from 7.4% at 3–5 years to 22.6% at ≥ 10 years of follow-up [2]. The high rates of LSG and the high rates of subsequent conversional would result in a considerably large volume of conversional procedures after LSG, raising attention to identifying the best conversional option with the best outcomes—studies of the safety and efficacy of different conversional options after LSG would be of value to decision-makers. RYGB is the most commonly reported conversional procedure after LSG, while other commonly reported conversional procedures than RYGB for SWL after LSG include re-sleeve, biliopancreatic diversion with duodenal switch (BPD-DS), single anastomosis duodenal-ileal bypass (SADI-S), and, less commonly, one-anastomosis gastric bypass (OAGB) [2, 6–9, 12]. Roux-en-Y gastric bypass (RYGB) as a conversional procedure has credible outcomes regarding weight loss, relief of GERD, and nutritional deficiencies [2, 6–9, 12]. The available literature includes multiple studies that have examined and compared the outcomes of various conversional procedures following LSG [2, 6, 9, 12, 13]; However, some studies have evaluated the results of conversional RYGB after LSG without making direct comparisons to alternative procedures [5, 7, 8, 10, 14–16], Furthermore, some studies have conducted comparisons between primary RYGB (PRYGB) cohorts and different cohorts of conversional RYGB following various types of previous operations [17]. In some cases, evaluating results became challenging due to the absence of stratification based on the specific procedure, resulting in a lack of direct evidence regarding the optimal procedure. Consequently, it was difficult to determine which procedure yielded the most favorable outcomes. In the literature, few studies have compared the outcomes of PRYGB to conversional RYGB following a single procedure, such as adjustable gastric band [18] or vertical banded gastroplasty [19]. To the best of our knowledge, there is a dearth of studies comparing the outcomes of primary Roux-en-Y gastric bypass (PRYGB) and conversional Roux-en-Y gastric bypass (CRYGB) exclusively following LSG. Consequently, this study aimed to assess and compare the short-term outcomes of matched cohorts undergoing uniform PRYGB and CRYGB procedures. This analysis of propensity score-matched cohorts may provide valuable insights for surgeons in selecting an appropriate conversational option for patients with suboptimal weight loss or who recurrenced weight after LSG.

## Methods

This retrospective cohort study analyzed data from records of patients who underwent PRYGB or CRYGB at three specialized bariatric centers between 2008 and 2019 and completed at least 2 years of follow-up. The study was approved by the Ethics Committee of the Medical Research Institute. Informed consent was obtained from all patients for the surgical procedure and the use of their data for research purposes.

### Study endpoints

The primary endpoints were weight loss and resolution of GERD. The secondary endpoints were food tolerance (FT), early and late complications, and the resolution of associated medical problems.

### Data collection

The collected data included patient demographics and associated medical problems; operative details such as operative time, combined procedures, and length of hospital stay; and pre-operative imaging and follow-up parameters.

For the CRYGB cohort, pre-operative collected data included the details of weight loss after the primary LSG, duration before the CRYGB, causes of conversional, and imaging and endoscopic findings.

### Pre-operative workup

All patients underwent routine abdominal ultrasound examinations to assess the need for concurrent cholecystectomy [20] and routine laboratory tests. Patients underwent routine pre-operative upper-endoscopy and multi-detector computed tomography (MDCT) virtual gastroscopy and volumetry. Gastroesophageal reflux disease (GERD) was classified according to the Los Angeles classification [21]. FT was assessed using a validated scoring system of 1–27 points; the higher the score, the better the FT [22]. For patients with suboptimal weight loss after the primary LSG was defined as nadir body mass index (BMI) > 35 kg/m^2^, and in patients who recurrence weight, it was defined as returning to a BMI of > 35 kg/m^2^ after decreasing to < 35 kg/m^2^ [23, 24] both revered in the text as SWL. Given the retrospective nature of our study and the available data in our database, we opted to use the cutoff value of BMI > 35 to define the best available selection criteria in the databases from our hospitals.

### Post-operative workup

All patients received anticoagulation prophylaxis with enoxaparin that started 12 h before surgery. A routine oral gastrografin test was performed on day 1. All patients for life were prescribed supplements with multivitamins and minerals, such as calcium and iron. Endoscopy was performed for persistent symptoms suggestive of GERD or marginal ulcers (MU) after medical treatment. MDCT with oral and intravenous contrast was performed when a leakage, bleeding, or intestinal obstruction was suspected.

### Follow-up parameters

Post-operative complications that occurred in the first 30 days (early) or later (late), readmissions, reoperations, and endoscopic findings were recorded. Weight loss, assessed by BMI, percentage of excess BMI loss (%EBMIL), percentages total weight loss (%TWL), and FT were assessed at 6 months and 1 and 2 years. The improvement and resolution of the associated medical problems were recorded at 2 years, according to the 2022 guidelines of the American Society of Metabolic and Bariatric Surgery (ASMBS) and the International Federation for the Surgery of Obesity and Metabolic Disorders (IFSO) [25] .

### Surgical techniques

The surgeons team performed all PRYGB and CRYGB procedures in all three clinics. Primary LSG was performed by this same team and partly referred from an external clinic (15% of the time). The surgical technique of PRYGB and CRYGB, our primary LSG technique, hiatal hernia repair, and concomitant cholecystectomy are described in Appendix 1.

### Statistical methods

For the analyses, we used descriptive and inferential statistics. All data were first tested for normality using the Kolmogorov-Smirnov test, Q-Q plot, and Levene’s test. Quantitative data were described as mean and standard deviation, while categorical data were summarized as frequency and percentage as appropriate. The chi-squared test (Χ^2^) was used to assess the significant association between two categorical variables. Fisher’s exact and the Monte-Carlo significance test were performed if more than 20% of total expected cell counts < 5. McNemar test was conducted for within-group differences. An independent sample t-test was performed to compare the mean quantitative variables between the two surgical interventions. A propensity score matching (PSM) analysis was performed using nearest neighbor matching and a ratio of 3:1 at caliper 1 to create a balanced sample of patients for CRYGB and PRYGB with adjustment for baseline covariates including age, sex, presence of associated medical problems, and pre-operative BMI. The average propensity score was statistically compared using the independent sample t-test and illustrated by a histogram plot to ensure a balanced distribution of propensity scores and proposed confounders between the group. Relative risk was calculated to compare the incidence of complications among patients who underwent CRYGB relative to standard PRYGB. A mixed design repeated measures analysis of variance (ANOVA) test was conducted to detect the main effects of post-operative time, the main effect of surgery, whether CRYGB or PRYGB and if an interaction was present in the form of a changing pattern of BMI, EBMIL%, %TWL along different post-operative periods between groups. A multiple linear regression model was conducted for entering the method to assess the independent contribution of surgery type adjusted for age, sex, and pre-operative BMI categorized as < 40 and ≥ 40 at 6 months, 1 year, and 2 years post-operative as the outcome variable. And a general estimation equation analysis was performed to produce unbiased average estimates with 95% CI of the BMI reduction among patients who underwent CRYGB and PRYGB at 2 years post-operative with adjustment for age, sex, and categorical pre-operative BMI (< 40 and ≥ 40). Stratification and subgroup analysis were created to correct effect modification from the primary LSG. Statistical significance was set at *p* ≤ 0.05 and was conducted using SPSS Statistics for Windows (version 28.0; IBM Corp., Armonk, NY, USA) and R studio (version 4.0.4).

## Results

This retrospective cohort study analyzed data from the records of patients treated in 2008–2019 and identified 558 PRYGB and 155 CRYGB patients who completed a minimum of 2 years of follow-up.

### Loss to follow-up

A total of 691 patients underwent PRYGB in 2008–2016, while 174 patients underwent CRYGB in 2008– 2019 at the three centers. Among them, 558 (80.8%) patients in the PRYGB and 155 (89.1%) patients in the CRYGB completed a minimum of 2 years of follow-up.

### Baseline characteristics

The mean age was 37.8 ± 11.1 and 42.9 ± 7.3 years in the PRYGB and CRYGB cohorts, respectively (*p* < 0.001). Women constituted 72.9% and 87.1% of the PRYGB and CRYGB cohorts, respectively (*p* < 0.001). The mean BMI was 47.65 ± 7.04 and 45.31 ± 7.37 kg/m^2^ for the PRYGB and CRYGB cohorts, respectively (*p* < 0.001). The mean operative time and hospital stay duration was significantly higher in the CRYGB than in PRYGB (94.96 ± 19.1 min and 2.22 ± 0.8 days vs. 42.621 ± 10.29 min and 2.02 ± 0.28 days, respectively; *p* < 0.001). The incidence of associated medical problems was higher in the PRYGB group (*p* = 0.036), whereas that of combined procedures LSG and CRYGB was more prevalent than in PRYGB (*p* < 0.001) (Table 1).

**Table 1:** Comparison of demographic and associated medical problems between patients performing conversional RYGB after LSG and patients performing primary RYGB before and after propensity score matching

	Before PSM		Sig.	After PSM		Sig.
	Conversional RYGB after LSG (*n* = 155)	Primary RYGB (*n* = 558)		Conversional RYGB after LSG (*n* = 149)	Primary RYGB (*n* = 332)	
Age(years)	42.99 ± 7.26	37.81 ± 11.09	< 0.001*	42.74 ± 7.07	40.73 ± 10.97	0.052
Female Sex	135(87.1%)	407(72.9%)	< 0.001*	129(86.6%)	283(85.2%)	0.699
Weight	124.15 ± 18.39	133.04 ± 26.09	< 0.001*	124.70 ± 18.14	128.67 ± 24.42	0.076
Height	1.66 ± 0.07	1.67 ± 0.09	0.086	1.66 ± 0.07	1.67 ± 0.10	0.208
Pre-operative BMI	45.31 ± 7.37	47.65 ± 7.04	< 0.001*	45.53 ± 7.30	46.08 ± 6.45	0.408
Total hospital stays (days)	2.22 ± 0.81	2.02 ± 0.28	< 0.001*	2.23 ± 0.83	2.01 ± 0.22	0.002*
Operative time (min)	94.96 ± 19.08	42.621 ± 10.29	< 0.001*	94.72 ± 19.22	42.48 ± 9.92	< 0.001*
**Preoperative associate medical problems**						
≥ 1 one problem	63(40.6%)	250(44.8%)	0.036*	59(39.6%)	140(42.2%)	0.569
IHD	3(1.9%)	53(9.5%)	0.002*			
Dyslipidemia	33(21.3%)	213(38.2%)	< 0.001*	30(20.1%)	120(36.1%)	< 0.001*
DM	18(11.6%)	56(10.0%)	0.048	16(10.7%)	33(9.9%)	0.789
HTN	25(16.1%)	50(9.0%)	0.010*	23(15.4%)	26(7.8%)	0.011*
**Combined surgery**						
No combined surgery	60(38.7%)	464(83.2%)	< 0.001*	56(37.6%)^b^	273(82.2%)^a^	< 0.001*
Cholecystectomy	10(6.5%)	51(9.1%)		10(6.7%)^a^	33(9.9%)^a^	
Hiatal hernia repair	61(39.4%)	31(5.6%)		61(40.9%)^b^	18(5.4%)^a^	
Cholecystectomy and hiatal hernia repair	24(15.5%)	12(2.2%)		22(14.8%)^b^	8(2.4%)^a^	

PSM: propensity score matching by nearest neighboring method, ratio 3:1. Different superscript letters denote significant pairwise comparison with adjusted significance. * Statistically significant results ≤ 0.05

### Propensity score matching

**Fig. 1:**
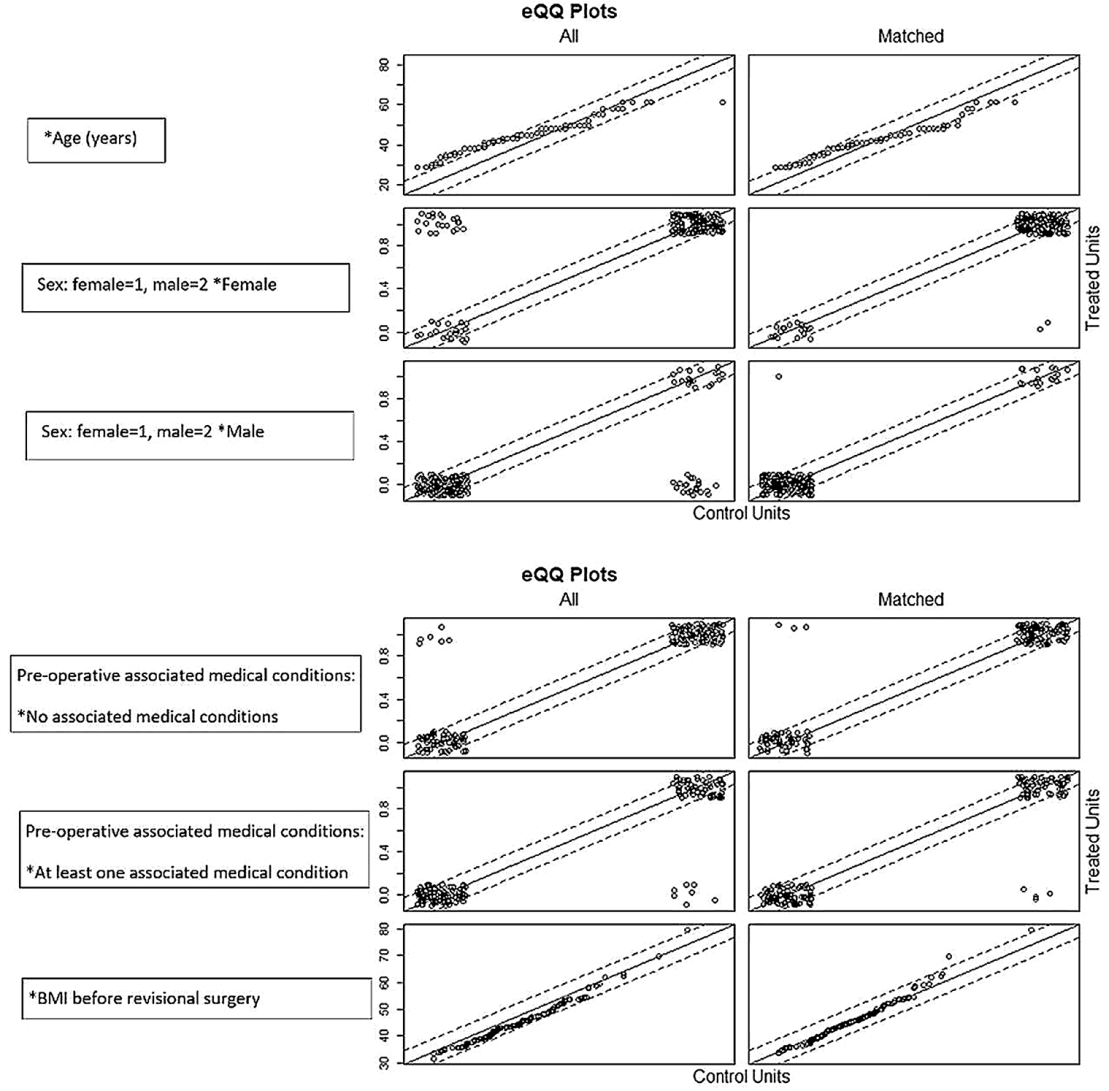
Distribution of propensity score by age, sex, BMI before conversion, and pre-operative associated medical problems covariates among patients performing Conversional RYGB after LSG and patients performing Primary RYGB before and after PSM. Treated, Conversional RYGB after LSG; Control, Primary RYGB

The PSM was tested by age, sex, BMI before conversional, and pre-operative associated medical problems covariates among the patients in both cohorts. After PSM, 149 patients in the CRYGB cohort and 332 patients in the PRYGB cohort were matched for comparable confounders: age (*p* = 0.052), sex (*p* = 0.699), pre-operative BMI (*p* = 0.408), and the presence of one or more associated medical problems (*p* = 0.569) (Fig. 1).

#### Pre-operative findings of the CRYGB cohort

The CRYGB cohort after PSM included 149 patients whose mean BMI before LSG was 49.11 ± 8.71 kg/m^2^, mean nadir BMI after LSG was 30.39 ± 5.9 kg/m^2^, and mean BMI before CRYGB was 45.53 ± 7.30 kg/m^2^. SWL was present in 31 (20.8%) patients, with recurrence weight in 105 (70.5%) patients.

Routine preoperative endoscopy in the CRYGB cohort revealed GERD in 122 (81.9%) patients: grade A in six (4.03%), grade B in 73 (48.9%), and grade C or D in 43 (28.9%). A sliding hiatal hernia was identified in 83 (55.7%) patients. The indication of conversion of LSG into CRYGB was SWL without GERD in 104 (69.8%) patients, symptomatic GERD without SWL in 13 (8.7%) patients, and both SWL and GERD in 32 (21.5%) patients; *p* < 0.001 (Table 2).

**Table 2:** Indications of conversion of LSG to RYGB

	Before PSM		After PSM	
Suboptimal weight loss without symptomatic GERD	107 (69%)	< 0.001*	104 (69.8%)	< 0.001*
Suboptimal weight loss with symptomatic GERD	34 (22%)		32 (21.5%)	
Intractable GERD without weight loss failure	14 (9%)		13 (8.7%)	

PSM: propensity score matching by nearest neighboring method, ratio 3:1. * Statistically significant results ≤ 0.05

#### BMI changes

After PSM, the mean pre-operative BMI was 46.08 ± 6.45 and 45.53 ± 7.30 kg/m^2^ in the PRYGB and CRYGB cohorts, respectively (*p* = 0.408). Throughout follow-up, the mean BMI in the PRYGB versus CRYGB cohorts was 36.6 ± 6.08 kg/m^2^ and 33.88 ± 6.11 kg/m^2^ at 6 months, 26.75 ± 5.54 kg/m^2^ and 29.34 ± 4.54 kg/m^2^ at 1 year, and 24.63 ± 3.47 kg/m^2^ and 28.39 ± 2.39 kg/m^2^ at 2 years, respectively.

**Fig. 2:**
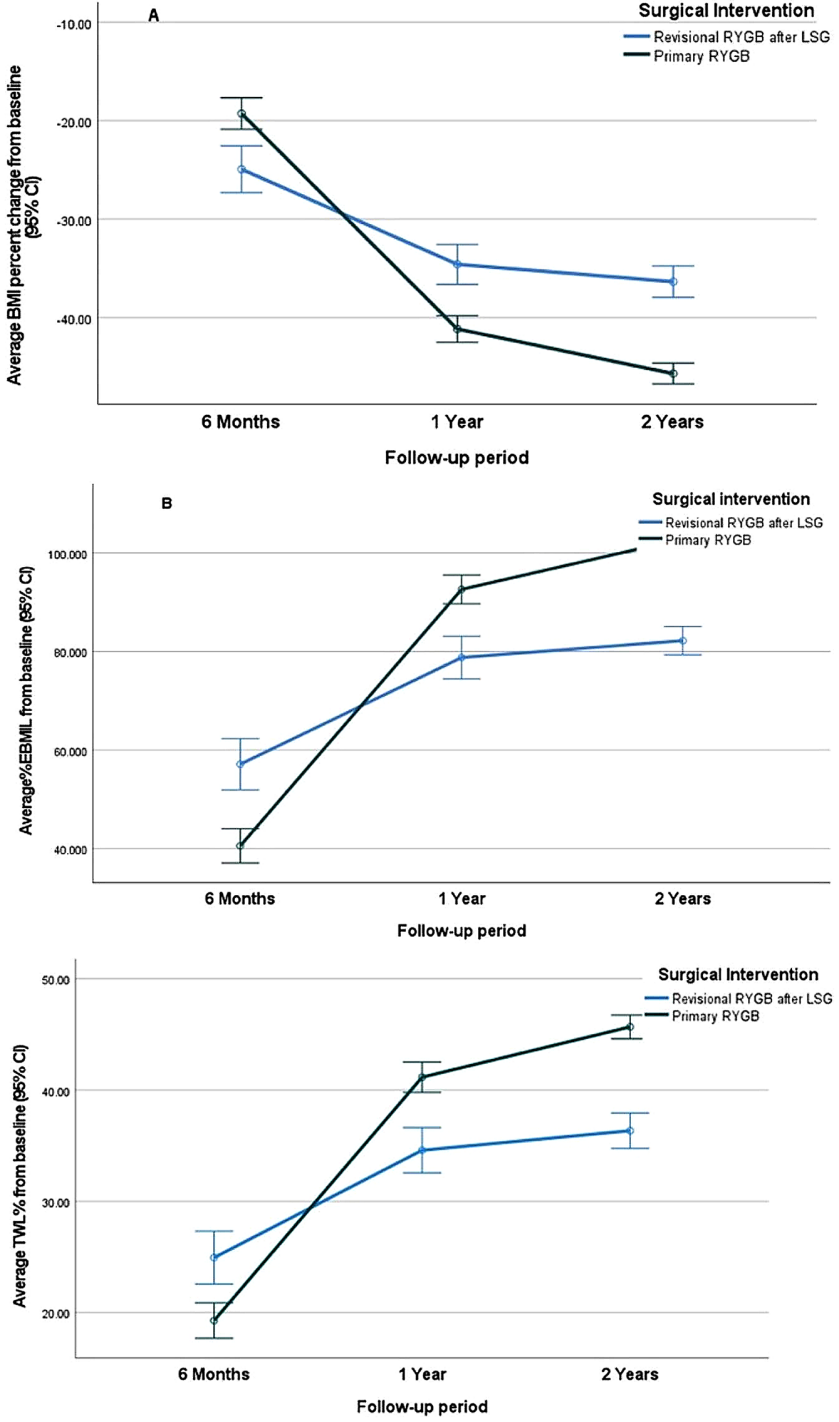
Error bar graph displaying results of mixed design repeated measures analysis of variance (ANOVA) test. (A) Average BMI percent change from baseline, (B) Average EBMIL% and, (C) Average TWL% from baseline with respective 95% confidence interval (CI) separately for Conversional RYGB after LSG and Primary RYGB groups after PSM

A significant overall decrease in mean BMI from baseline throughout the follow-up period was detected in both cohorts (*p* < 0.001) (Fig. 2; Table 3). The PRYGB cohort showed a significantly greater decrease in mean BMI and a more tremendous increase in mean %EBMIL and %TWL than the CRYGB cohort after 1 and 2 years (*p* < 0.001).

**Table 3:** Mixed design repeated measures analysis of variance (ANOVA) test comparison of average BMI percent change from baseline, average EBMIL%, and average %TWL at different follow-up periods for conversional RYGB after LSG and primary RYGB groups after PSM

	Conversional RYGB after LSG (*n* = 149)	Primary RYGB (*n* = 332)	Sig.
**% Change BMI from baseline**			
6 months 1Yr 2Yr	-24.94 ± 11.60	-19.28 ± 15.97	p^a^ <0.001* p^b^<0.001* p^c^<0.001*
	-34.59 ± 11.14	-41.16 ± 13.25	
	-36.35 ± 9.91	-45.68 ± 9.87	
**%EBMIL**			
6 months 1Yr 2Yr	57.12 ± 26.61	40.55 ± 34.51	p^a^ <0.001* p^b^.001* p^c^<0.001*
	78.76 ± 22.43	92.60 ± 28.82	
	82.19 ± 14.22	102.86 ± 19.30	
**%TWL**			
6 months	24.94^a^ ± 11.60	19.28^a^ ± 15.97	p^a^ <0.001*
1Yr	34.59^b^ ± 11.14	41.16^b^ ± 13.25	p^b^.001*
2Yr	36.35^c^ ± 9.91	45.68^c^ ± 9.87	p^c^<0.001*

Mixed design repeated measures ANOVA test to assess the main **effect of time**, ^a^ main **effect of surgical interventions**, ^b^ and interaction to assess the **pattern of change** of each quantitative variable along time by surgical intervention, ^c^ *Significant results ≤ 0.05

SWL 2 years after CRYGB was recorded in three (2.0%) patients in the CRYGB cohort versus 31 (20.8%) patients in the PRYGB cohort.

#### Multivariate analysis of BMI outcomes

After adjustment for surgical intervention type, age, sex, and pre-operative BMI, the PRYGB cohort showed an initial significantly lower BMI decrease compared to the CRYGB cohort at 6 months after PSM (6 months: coeff. 6.095 kg/m^2^; [95% CI, 3.469–8.721]; *p* < 0.001), while at 1 and 2 years, the PRYGB cohort showed a significantly higher mean BMI decrease compared to the CRYGB cohort (1 year: coeff. -5.890 kg/m^2^; [95% CI, -8.165 to -3.614]; *p* < 0.001; 2 years: coeff. -8.626 kg/m^2^; [95% CI, -10.252 to -6.999]; *p* < 0.001), respectively (Table 4).

**Table 4:** Multivariate linear regression analysis and general estimation equation for predicting BMI percent change from baseline at 6 months, one year and two years adjusted for type of surgery, age, sex, and preoperative body mass index before and after PSM

Covariates	% BMI change from baseline					
	Before PSM			After PSM		
	Coeff.	[95% CI]	Sig.	Coeff.	95% CI	Sig.
**At 6 months**: †						
Primary RYGB	5.048	[2.470 to7.626]	< 0.001*	6.095	[3.469 to 8.721]	< 0.001*
Female	− 0.580	[-2.998 to1.839]	0.638	-1.373	[-4.870 to 2.123]	0.441
Age in years	− 0.091	[-0.190 to.007]	0.070	− 0.232	[-0.356 to − 0.109]	< 0.001*
Preoperative BMI ≥ 40	-16.101	[-18.89 to -13.305]	< 0.001*	-13.808	[-16.898 to -10.718]	< 0.001*
**At 1 year**: †						
Primary RYGB	-6.849	[-9.027 to-4.672]	< 0.001*	-5.890	[-8.165 to -3.614]	< 0.001*
Female	− 0.391	[-2.434 to1.652]	0.707	− 0.443	[-3.473 to 2.588]	0.774
Age in years	0.007	[-0.077 to 0.090]	0.876	− 0.069	[-0.176 to 0.038]	0.207
Preoperative BMI ≥ 40	-13.888	[-16.24 to -11.526]	< 0.001*	-12.284	[-14.962 to -9.606]	< 0.001*
**At 2 years**: †						
Primary RYGB	-9.948	[-11.531 to-8.366]	< 0.001*	-8.626	[-10.252 to -6.999]	< 0.001*
Female	− 0.187	[-1.672 to1.297]	0.804	− 0.554	[-2.719 to 1.611]	0.615
Age in years	0.005	[-0.055 to.066]	0.859	− 0.082	[-0.159 to -0.005]	0.036*
Preoperative BMI ≥ 40	-14.465	[-16.18 to-12.749]	< 0.001*	-13.140	[-15.053 to -11.227]	< 0.001*
**General estimation equation regression analysis**: ††						
	**Est. Average**	**[95% CI]**	**Sig.**	**Est. Average**	**[95% CI]**	**Sig.**
**Type of surgery**:						
Conversional RYGB after LSG	-27.974	[-29.402 to -26.545}	< 0.001*	**-**27.701	[-29.372to -26.030]	< 0.001*
Primary RYGB	-31.891	[-32.990 to -30.792]		-30.487	[-32.158 to-28.816]	
**Preoperative BMI**:						
< 40	-22.523	[-24.216 to -20.831]	0.001*	-22.532	[-24.654 to -20.412]	0.001*
≥ 40	-37.341	[-38.273 to -36.409]		-35.655	[-37.039 to-34.270]	

*Significant results ≤ 0.05

†Data indicate change from baseline at 6-months, 1-year, and 2-years. Coeff, Coefficients of the multiple linear regression analysis go along with 95% confidence intervals (CI), predictors are age, sex, preoperative BMI, and type of surgery

††General estimation equation to predict BMI change from baseline at 2 years postoperative follow-up period adjusted for age, sex, preoperative BMI, and type of surgery. Sex was insignificant predictor of % BMI change from baseline at 6months, 1 year and 2 years follow-up before and after PSM

The general estimation equation regression analysis showed a significantly higher estimated BMI decrease from baseline in the PRYGB cohort after PSM (2 years: coeff. -30.487; [95% CI, -32.158 to -28.816]) versus the CRYGB cohort (2 years: coeff. -27.701; [95% CI, -32.158 to -28.816]), (*p* < 0.001) (Table 4). Moreover, patients with pre-operative BMI ≥ 40 had a significantly higher estimated BMI drop from baseline after PSM (2 years: coeff. -35.655; [95% CI: -37.039 to-34.270]) compared to patients with pre-operative BMI < 40 (2 years: coeff. -22.532; [95% CI: -24.654 to -20.412]) (p = < 0.001) (Table 4).

### Food tolerance

The mean pre-operative FT score in the CRYGB cohort (22.07 ± 0.84) significantly increased to 23.98 ± 0.78 at 2 years of follow-up (*p* < 0.001). At 2 years of follow-up, the mean FT score in the PRYGB cohort was 23.05 ± 0.78, significantly lower than the FT score of the CRYGB cohort (*p* < 0.001).

### GERD resolution

At 2 years after CRYGB, only 11 (7.4%) patients had residual GERD; all cases were grade A (*p* < 0.001).

### Complications

The cohorts showed a comparable incidence of complications with recorded rates of 4.7% versus 5.7% for early complications (*p* = 0.584) and 4.7% versus 5.1% (*p* = 0.495) for late complications in the CRYGB and PRYGB cohorts. One case (0.7%) of leakage was recorded in the CRYGB cohort versus none in the PRYGB cohort. The leak was successfully treated using a self-expandable metallic stent (SEMS). Intra-abdominal bleeding was recorded in two (1.3%) cases in the CRYGB group versus five (1.5%) cases in the PRYGB group (Table 5).

**Table 5:** Comparison of outcomes (early, late complications, readmission, and reoperation) between conversional RYGB after LSG and primary RYGB cohorts after PSM

		Primary RYGB		Conversional RYGB after LSG		Sig.
		N (332)	%	N (149)	%	RR [95% CI]
**Early complications**	MVO	4	1.2%	1	0.7%	^MC^p.584
	Melena	8	2.4%	1	0.7%	
	Bleeding	5	1.5%	2	1.3%	
	Leak	0	0.0%	1	0.7%	
	Intestinal obstruction/hernia	1	0.3%	1	0.7%	
	Wound infection	1	0.3%	1	0.7%	
**Late complications**	Marginal ulcer	13	3.9%	4	2.7%	^MC^p.495
	Port site hernia	4	1.2%	2	1.3%	
	Internal hernia	0	0.0%	1	0.7%	
**Readmission**	No	325	97.9%	139	93.3%	^FE^p.011*
	Yes	7	2.1%	10	6.7%	0.3142 [0.12 to 0.80]
**Reoperation**	No	324	97.6%	140	94.0%	^FE^p.053
	Yes	8	2.4%	9	6.0%	0.398 [0.157 to 1.013]

RR: relative risk compares incidence of complication among patients underwent conversional RYGB after LSG relative to standard Primary RYGB standard approach, (95% CI): 95% Confidence interval, ref: reference category. ^MC^ Monte-Carlo significance. ^FE^ Fischer-Exact significance

Venous mesenteric vascular occlusion (MVO) was reported in one (0.7%) CRYGB patient versus four (1.2%) PRYGB. Within the first 3 weeks post-operative, it presented as persistent epigastric pain, a low-grade fever, and leukocytosis. An MDCT scan of the abdomen with intravenous contrast confirmed the diagnosis. Conservative treatment with anticoagulants and fluid resuscitation was successful in patients of the PRYGB cohort, while the CRYGB patient required limited resection of a short loop of the jejunum.

An internal hernia was recorded in one (0.7%) patient in the CRYGB group and one (0.3%) patient in the PRYGB, with herniation of the bowel occurring through mesenteric defects. Both patients presented with intestinal obstruction at 3–4 weeks post-operative and were managed with laparoscopic reduction of the hernia and repair of the defect.

Melaena occurred in eight (2.4%) PRYGB patients versus one (0.7%) CRYGB patient. All patients succeeded in conservative management by discontinuing anticoagulants, fluid resuscitation, and blood transfusion (Table 5).

MU was reported in 13 (3.9%) and four (2.7%) patients in the PRYGB and CRYGB cohorts, respectively. The main presentation was persistent epigastric pain, vomiting, and melena. All patients were smokers, and some used nonsteroidal anti-inflammatory drugs excessively. Conservative management was successful in all patients.

Port site hernias occurred in four (1.2%) and two (1.3%) patients in the PRYGB and CRYGB cohorts. Surgical repair was successfully performed in all patients (Table 5).

Readmissions were recorded in seven (2.1%) and 10 (6.7%) patients in the PRYGB and CRYGB cohorts, respectively (*p* = 0.011). Dehydration, MVO, vomiting, and melena were the most common causes of readmission (Table 5).

### Associated medical problems

Both cohorts showed significant improvement from baseline in the overall associated medical problems at 2 years after RYGB (*p* < 0.001) (Appendix 2).

**Fig. 3:**
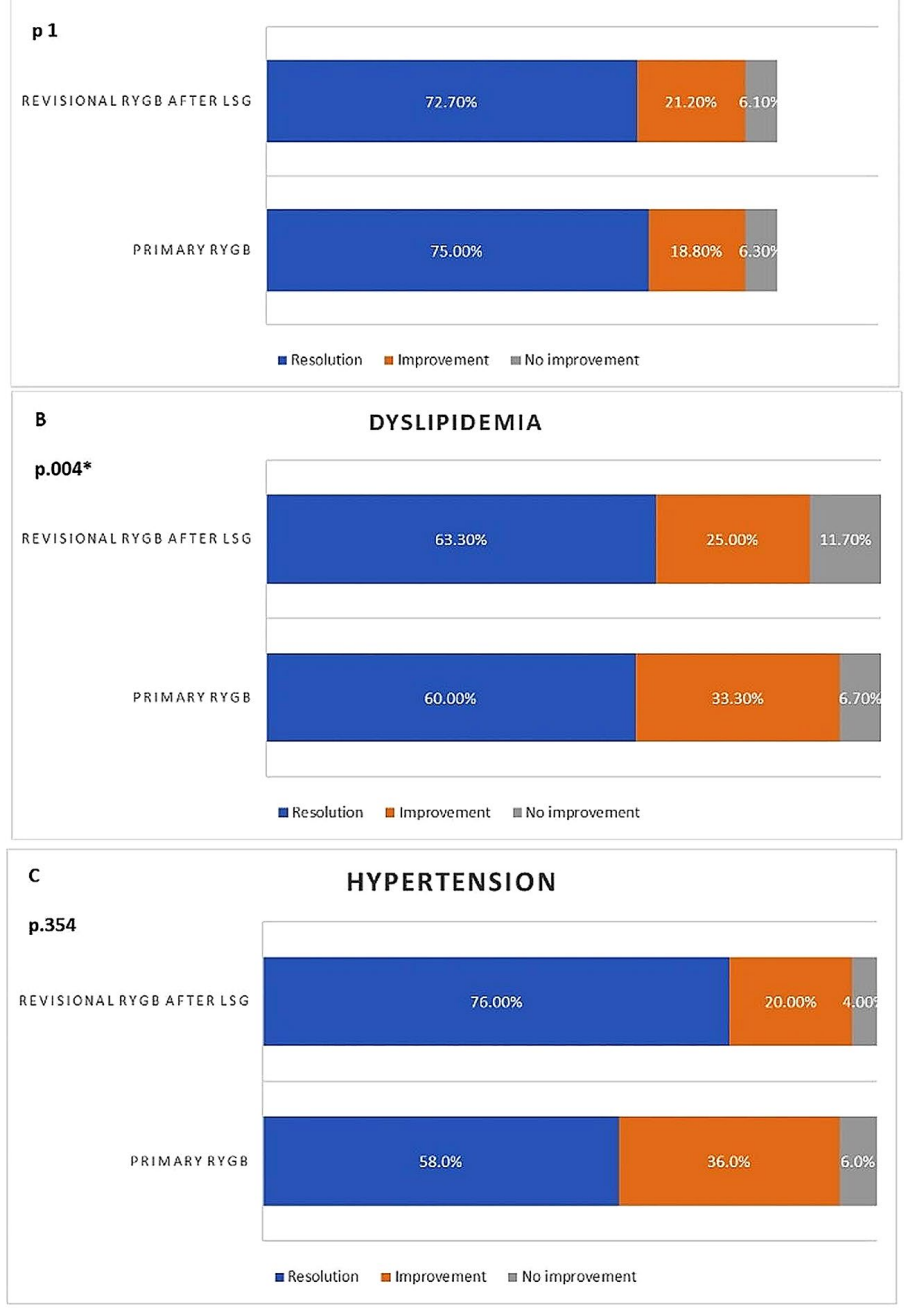
Comparison of proportion of associated medical problems (A) DM, (B) Dyslipidemia, and (C) Hypertension between preoperative and 2-years follow-up period; separately for Conversional (revisional) RYGB after LSG (*n* = 149), B) Primary RYGB group (*n* = 332) after PSM

Rates of type 2 diabetes mellitus resolution were 72.7% and 75.7%, and the rates of improvement were 21.2% and 18.7% in the CRYGB and PRYGB cohorts, respectively (*p* = 1). Rates of hypertension resolution were 58.0% and 76.0%, rates of improvement were 36.0% and 20.0% in the CRYGB and PRYGB cohorts, respectively (*p* = 0.354). Rates of dyslipidemia resolution were 63.43% and 60.2%, rates of improvement were 24.9% and 33.3% in the CRYGB and PRYGB cohorts, respectively (*p* = 0.004) (Fig. 3).

## Discussion

This was a retrospective cohort trial with a propensity scoring matching between PRYGB and CRYGB with SWL after primary LSG.

To the best of our knowledge, the CRYGB cohort in this study is the largest reported cohort of RYGB after LSG to date in the literature. Initially, it included 155 CRYGB patients who operated between 2008 and 2019 and completed 2 years of follow-up. After PSM, 149 patients were included in the CRYGB cohort, matched with 332 patients in the PRYGB cohort.

### Weight loss

In this study, both cohorts experienced a significant mean BMI decrease after RYGB (*p* < 0.001); however, the PRYGB cohort had a significantly higher reduction in mean BMI and higher mean %EBMIL than the CRYGB cohort (*p* < 0.001). The lower weight loss efficiency of CRYGB than PRYGB is a common finding in the literature.

A meta-analysis of studies comparing PRYGB and CRYGB outcomes showed significantly inferior weight loss outcomes in CRYGB in 10 studies and non-significant weight loss differences in two studies, all of which had various CRYGB cohorts, including patients with different primary bariatric procedures [17].

Moreover, studies comparing PRYGB cohorts to similar cohorts of CRYGB following one primary procedure, for example, adjustable gastric banding or vertical banded gastroplasty, also showed less weight loss with CRYGB [18, 19].

Although the CRYGB cohort had less of a BMI decrease versus the PRYGB cohort in this study and in the literature, the CRYGB still had better weight loss compared to the initial LSG, with only three (2%) cases of SWL after CRYGB versus 31 (20.8%) cases after PRYGB (*p* < 0.001). This is in line with the published data in the literature denoting better weight loss after combining two surgeries (primary plus conversional versus primary surgery alone) [17].

So, a good decision in what the best primary surgery would be, with the option for conversional in some instances, is essential when SWL is expected in the first stage due to multiple reasons, whereby a conversional surgery can help a patient give a back-to-life opportunity after SWL after the first attempt with BMS.

### Conversion surgery

LSG is easily convertible to almost any other BMS, compared to other BMS procedures with fewer conversional options. While the best conversional procedure choice remains debated, some recommendations are available in the literature: re-sleeve may be preferred in cases of SWL with sleeve dilatation in the absence of associated medical problems. In contrast, SWL with no significant dilatation should benefit from added malabsorption as biliopancreatic diversion with duodenal switch and single anastomosis duodenal-ileal bypass with Sleeve or conversion to a gastric bypass procedure such as RYGB and one-anastomosis gastric bypass. Furthermore, RYGB may still be the preferred procedure for GERD after LSG (2). This study’s main indication for conversion from LSG to CRYGB was SWL alone (58.4%), followed by SWL with GERD (32.9%). When we look at the literature, SWL and GERD are the main reported indications for conversional surgery after LSG [2, 8–10]. Other reported indications for conversion included persistent dysphagia due to stenosis or torsion of the sleeve pouch [9, 15].

In a recent systematic review, SWL accounted for 52.0% of conversional after LSG, while GERD accounted for 30.4% of conversional [10]. Despite the initial excellent weight loss, SWL remains a long-term problem after LSG, with a high incidence of up to 75.6% in systematic reviews and meta-analyses [26, 27]. Causes of SWL include pouch dilatation, non-compliance to the required diet, and behavioral modifications [2, 27]. Thus, psychological and behavioral support are essential for multidisciplinary follow-up after LSG.

### Complications

CRYGB showed a similar safety profile to PRYGB in this study, with a comparable incidence of early (*p* = 0.584) and late (*p* = 0.495) complications. CRYGB has a reported safety profile comparable to PRYGB, with non-significant differences in the rates of complications, mortality, and re-operation [8, 16, 17]. Both cohorts in this study had a relatively low incidence of complications (9.4% vs. 10.8% overall complication rates and 6% vs. 2.4% re-operation rates in the CRYGB and PRYGB groups, respectively), with no cases of mortality in either cohort. Data from systematic reviews reported overall complication rates of 29.5% vs. 13.9%, 8.4% vs. 8.6% re-operation rates, and 1.3% vs. 0.2% mortality rates in the CRYGB and PRYGB groups, respectively [17]. Another recent review reported early and late complication rates of 16.4% and 11.4%, respectively, after CRYGB [10].

### Leaks

Leaks are reported complications after all BMS, with a higher incidence among conversional procedures. CRYGB has a reported leak rate of 5.8% (vs. 1% for PRYGB) in the systematic review [17]. In this study, the leak occurred in one case (0.7%) in the CRYGB vs. no cases in the PRYGB and was successfully managed by SEMS. Leaks after RYGB can be safely managed using SEMS [28].

### Marginal ulcers

The incidence of MU was slightly lower in the CRYGB (2.7%) versus PRYGB (3.9%) in this study, with no statistical difference (*p* = 0.495). There is no clear relationship between the incidence of MU and the nature of the procedure, either primary or conversional, but data from a recent multi-institutional study demonstrated a significantly higher incidence of MU for CRYGB than PRYGB [29].

### Readmissions

The CRYGB cohort had a significantly higher incidence of readmission than the PRYGB cohort (6.7% vs. 2.1%, respectively; *p* = 0.011). Persistent vomiting and dehydration, melena, and MVO were the leading causes of readmission. A higher incidence of readmission due to intolerance to oral feeding or other complications is common after CRYGB [30].

### GERD

Data from a recent systematic review showed de novo GERD in 9.3% and 2.3% after LSG and RYGB, respectively, with LSG featuring a significantly higher risk of GERD versus RYGB; however, GERD improvement occurred in 40.4% versus 74.2% in patients with obesity and with GERD having LSG or RYGB, respectively, with RYGB having significantly higher remission rates of GERD [31].

A significant GERD resolution was noted in this study for the CRYGB cohort; resolution of GERD happened in 91% of the patients at 2 years of follow-up (*p* < 0.001). No cases of de novo GERD was recorded in this study.

The conversion from LSG to RYGB has a reported excellent potential for resolution of GERD with > 90% complete or partial resolution of GERD [2, 8, 10, 14, 16, 32, 33]. Even in the absence of significant weight loss after CRYGB, significant resolution of GERD was achieved, as reported in some studies [34]. CRYGB should be considered in patients treated with primary LSG and GERD refractory to medical treatment.

### Food tolerance

The CRYGB cohort experienced significant improvement in mean FT scores after the conversional surgery (*p* < 0.001). Moreover, the CRYGB cohort after LSG had a significantly higher mean FT score than the PRYGB cohort after 2 years (*p* < 0.001). The lower FT scores in the PRYGB group could be correlated with the effect of the BMS with restriction and dumping. The CRYGB cohort exhibited improved FT scores, although LSG reportedly had better FT scores than RYGB [35]. This may be attributed to the high incidence of GERD in the PRYGB cohort, which dramatically improved after CRYGB. Food tolerance scores primarily reflect a patient’s perceived ability to eat and enjoy a variety of foods without discomfort or symptoms like nausea, vomiting, or abdominal pain. On the other hand, conditions like dehydration, MVO, vomiting, and melena may arise from the altered gastrointestinal anatomy post-RRYGB, independent of subjective food tolerance.

## Associated medical problems

This study’s CRYGB and PRYGB cohorts significantly improved from baseline in overall associated medical problems at 2 years after RYGB (*p* < 0.001). Both groups significantly improved regarding the associated medical problems in the literature [17]. Moreover, in some studies, CRYGB is significantly associated with medical problems, even without additional weight loss benefits [8]. Therefore, conversional surgery provides additional health benefits that should be discussed when a patient consults a physician.

## Role of correcting for confounding factors

Comparing outcomes between primary RYGB and conversional RYGB following LSG can be seen as a very different group of patients, with potential selection bias. Still, we need to pay attention to this methodical phenomenon and not ignore it. As researchers, it is crucial to explore the effects of different interventions and procedures to understand their impact on patient outcomes better. Comparing the outcomes of patients who have undergone CRYGB after being identified as SWL following LSG with those who have undergone primary RYGB provides valuable insights into the potential effects of the conversional procedure itself, as well as other contributing factors. To manage these confounding variables, we employed PSM, a statistical technique that helps to reduce bias in observational studies. This technique effectively compensates for potential confounding variables that could skew the results of our analysis. It allowed us to create two comparable groups across a wide range of observed characteristics for a better-balanced outcome of the results.

## Limitations

This study included large cohorts of CRYGB and PRYGB treated with the same surgeons, crediting the uniformity of the data; however, some limitations existed. The retrospective design may be inferior to the prospective randomized design because of selection bias. Nonetheless, randomization is not applicable between conversional and primary cases. Endoscopic examinations were not performed routinely post-operatively for all patients; instead, they were selectively conducted for those presenting symptoms. This approach may have led to an underestimation of the incidences of GERD and MU. It’s noteworthy that a considerable number of patients display upper gastrointestinal irregularities during EGD prior to BMS, including those without symptoms. Although certain abnormalities detected may not alter the course of medical or surgical treatment, performing routine preoperative EGD is advisable and can be determined based on the surgeon’s judgment. It should be noted that the American Society for Metabolic and Bariatric Surgery (ASMBS) guidelines were published after the timeframe of our study and were not known at that time [36]. Regarding data on weight loss after primary LSG was incomplete, since part of the patients were referred by another clinic, that data was unavailable. The data on weight loss in primary LSG from our clinic was after propensity score matching (PMS) inconsistent present in the selected group and therefore low in power and excluded. Despite the previously mentioned benefit of PMS creating matched cohorts based on specific covariates; however, some covariates may be missed, leading to some degree of imbalance between the study cohorts. Moreover, PSM may exclude patients with essential findings from the new smaller samples.

## Conclusion

CRYGB is a safe and effective option in SWL or GERD after LSG. CRYGB had comparable rates of complications, reoperations, and associated medical problem resolution to those of PRYGB. CRYGB resulted in significant weight loss and improved GERD. The overall weight loss after CRYGB was significantly lower than that after PRYGB but was still significant and satisfactory. Moreover, CRYGB had significantly lower SWL rates than previous LSG. FT in the CRYGB group improved significantly after CRYGB and was significantly better than that after PRYGB.

### Electronic supplementary material

Below is the link to the electronic supplementary material.

Supplementary Material 1: Surgical workflow per procedure

Supplementary Material 2: Comparison of different associated medical problems between patients performing Revisional RYGB after LSG and patients performing Primary RYGB at preoperative and 2-year follow-up periods

## Data Availability

The datasets used and/or analyzed during the current study are available from the corresponding author upon reasonable request.
