# Supplementary material for: Outcomes of primary versus conversional Roux-En-Y gastric bypass after laparoscopic sleeve gastrectomy: a retrospective propensity score–matched cohort study
Source: BMC Surg. 2024 Mar 6;24:84. doi: 10.1186/s12893-024-02374-7 (PMC10919008; doi:10.1186/s12893-024-02374-7)
Supplement: Supplementary file 1 — Supplementary Material 1: Surgical workflow per procedure [file 12893_2024_2374_MOESM1_ESM.docx]

**Appendix 1: Surgical workflow per procedure**

**Revisional RYGB from LSG**

Adhesions were dissected around the gastric sleeve using the energy device EnSeal^®^ (Ethicon Endo-Surgery, Cincinnati, OH, USA). The gastric pouch was created from 5–6 cm below the esophagogastric junction using an Echelon Flex Endopath 60-mm linear stapler (Ethicon Endo-Surgery, Cincinnati, OH, USA) over a 40 Fr bougie, with gold and blue reloads. The same stapler was used to constructing the gastro-jejunostomy and jejuno-jejunostomy using blue and white reloads, respectively, with equal 100 cm biliopancreatic and alimentary limbs. The stapling defects were closed in two layers using barbed sutures (3/0 V-Loc 180 sutures [Covidien, Mansfield, MA, USA]). The staple line in the gastric pouch and remnant stomach was reinforced with seromuscular continuous sutures using the same barbed sutures. All mesenteric defects were closed using 3/0 V-Loc non-absorbable sutures (Covidien, Mansfield, MA, USA).

**Primary RYGB**

A pneumoperitoneum was created using visual entry trocars and a 0^o^ angled lens; five standard ports were used, including three 12-mm ports (for the camera, and right and left working ports) and two 5-mm ports (for liver retraction and the assistant). The pouch was created with the same linear stapler using gold and blue reloads. The lengths of the biliopancreatic and alimentary limbs were equal, 100 cm each; as per revisional RYGB. The gastro-jejunostomy and the jejuno-jejunostomy were performed similarly to revisional RYGB.

All staple lines were reinforced with the same technique used for revisional RYGB. Mesenteric defects were also routinely closed. Intraoperative endoscopy was not routinely performed for primary RYGB. The blue dye leak test was routinely performed for the gastro-jejunostomy. A drain was routinely inserted in the left sub-phrenic space.

**Primary sleeve gastrectomy (PLSG):**

PLSG were performed via five ports—two 12-mm trocars and three 5-mm trocars—following the same technique; the greater omentum was dissected off, the greater curvature of the stomach up to the left crus of the diaphragm and down to approximately 2–4 cm from the pylorus, followed by dissection of any posterior gastric adhesions and excision of Belsey’s pad of fat, using the Enseal device (Ethicon Endosurgery, Cincinnati, OH, USA).

We used a 40-Fr bougie for calibration in all patients and Echelon Flex Endopath 60-mm linear stapler (Ethicon Endosurgery, Cincinnati, OH, USA) for gastric division, starting at 3–5 cm before the pylorus, up to the angle of His.

We start gastric stapling using black or green reloads according to the thickness of the antrum, then gold reloads for the body and blue reloads for the fundus compression time of 15 seconds. Ultimately, we applied running seromuscular stitches using unidirectional absorbable 3/0 V-Loc 180 sutures (Covidien, Mansfield, MA, USA) to invaginate the staple line completely.

**Hiatal hernia repair and concomitant cholecystectomy**

Closure of the intercrural defect using a continuous barbed prolene 2-0 suture (Covidien, Mansfield, MA). Narrowing of esophageal hiatus by barbed prolene 2-0 suture (Covidien, Mansfield, MA). Attaching the lateral part of the esophagus and the upper part of the stomach to the left crus. Concomitant cholecystectomy was performed in cases with pre-operatively diagnosed calcular cholecystitis without additional trocar ports.
