# Supplementary material for: Outcomes of primary versus conversional Roux-En-Y gastric bypass after laparoscopic sleeve gastrectomy: a retrospective propensity score–matched cohort study
Source: BMC Surg. 2024 Mar 6;24:84. doi: 10.1186/s12893-024-02374-7 (PMC10919008; doi:10.1186/s12893-024-02374-7)
Supplement: Supplementary file 2 — Supplementary Material 2: Comparison of different associated medical problems between patients performing Revisional RYGB after LSG and patients performing Primary RYGB at preoperative and 2-year follow-up periods [file 12893_2024_2374_MOESM2_ESM.docx]

**Appendix 2: Comparison of different associated medical problems between patients performing Revisional RYGB after LSG and patients performing Primary RYGB at preoperative and 2-year follow-up periods.**

| Associated medical problems | Pre-operative | At 2 Years | Sig. |
| --- | --- | --- | --- |
|  |  |  |  |
| Diabetes Mellitus: |  |  |  |
| Revisional RYGB after LSG | 16(10.7%) | 4(2.7%) | <.001* |
| Primary RYGB | 33(9.9%) | 9(2.7%) | <.001* |
| Dyslipidemia: |  |  |  |
| Revisional RYGB after LSG | 30(20.1%) | 12(8.1%) | <.001* |
| Primary RYGB | 120(36.1%) | 44(13.3%) | <.001* |
| Hypertension:‎ |  |  |  |
| Revisional RYGB after LSG | 24(16.1%) | 5(3.35%) | <.001* |
| Primary RYGB | 30(9.03%) | 11(3.31%) | <.001* |

Percentage of total patients undergoing Revisional RYGB after LSG intervention (n=149 after PSM) and Primary RYGB intervention (n=332 after PSM). Positive associated medical problems at 2 years include persistent and improved but not entirely resolved problems, while completely resolved problem was considered negative associated medical problems *Significant results≤.05
